# Pattern of Kebab Intake as a Potential Carcinogenic Risk Factor in Adults of Kermanshah, Iran: 2015

**Published:** 2018-01-01

**Authors:** Seyyed Mostafa Nachvak, Mahboobe Hosseinikia, Hadi Abdollahzad, Yahya Pasdar, Farhad Oubari, Roghaye Hosseinikia, Maryam Shabanpur

**Affiliations:** 1 Department of Nutrition, Faculty of Public Health, Kermanshah University of Medical Sciences, Kermanshah, Iran; 2 Medical Biology Research Centre, Kermanshah University of Medical Sciences, Kermanshah, Iran; 3 Department of Health Education and Nutrition, Faculty of Public Health, Kermanshah University of Medical Sciences, Kermanshah, Iran

**Keywords:** Kebab, Dietary pattern, Cancer

## Abstract

**Background: **Epidemiologic studies indicated that dietary pattern plays a determinant role in cancer incidence. They also indicated that 1/3 of cancers are associated to foods. Diet contains different carcinogenic agents: naturally occurring chemicals, synthetic components and compounds produced during cooking such as kebab. This traditional food is one of the most popular foods in the Middle East, particularly in Iran. Red meat, especially lamb or veal, is the most common meat used in preparation of kebab. Since kebab is considered as a food containing carcinogenic compounds, so the purpose of this study was to assess the consumption pattern of kebab in a sample of Iranian adults and its relationship with demographic characteristics.

**Materials and Methods:** This cross-sectional study was conducted between March and April 2015 on 705 Iranian adults who were living in Kermanshah province in the west of Iran. Subjects were selected randomly from different districts of Kermanshah. Data were collected through a questionnaire survey which had been designed by academic members of Department of Nutrition at Kermanshah University of Medical Sciences. Data analysis was performed using SPSS Version 20. The results were expressed as mean ± SD. Student’s t-test, ANOVA and chi-square tests were performed to compare the study groups. The normality of data was assessed using the Kolmogorov-Smirnov test. All results were analyzed using a significance level of P <0.05.

**Results: **The results indicated that nearly 60% of subjects have a high tendency to consume kebab. The average of kebab consumption among the participants in this study was 4 times per month. Nearly, 85% of study participants tended to consume kebab with a large amount of salt. The chi-square test determined the significant difference between education and tendency to consume kebab; individuals with higher level of education had more tendency to consume kebab than those having lower level of education (p=0.021). In this study, 93.9% of participants used charcoal, a cooking fuel, to prepare kebab.

**Conclusion: **The results of this study point out that the study participants, regardless of socio-economic status, consume high amounts of kebab, and thus this unhealthy eating habit will increase the risk of carcinogenesis. Therefore, the immediate attention of Public Health Officials is required.

## Introduction

 Cancer is one of the major public health problems in Iran. According to the latest statistical and epidemiological studies in Iran, after cardiovascular disease and unintentional injuries, cancer is the third leading cause of death. Over 30000 people die of the disease annually, and it is estimated that more than 70,000 new cases of cancer deaths will occur each year in the country. Cancer incidence rate in Iran is 135 in 100000 per year. The increase in life expectancy and the aging of population will sharply rise the incidence of cancer in the next decade^1^

 Many epidemiologic studies, both in human and in animal models, have suggested that dietary pattern plays an important role in cancer incidence. Epidemiologic studies indicated that 1/3 of cancers are associated to foods^2^.

Cancer is a common expression which describes a condition in which cells divide without control and these abnormal cells can spread to different parts of the body by blood or lymphatic system. Cancer is best-known cause of deaths worldwide, with nearly 14 million new cases and 1.8 million deaths in the year^3^^, ^^4^.

Diet contains different carcinogenic agents: naturally occurring chemicals, synthetic components and compounds produced during cooking. In the last decades, researchers have focused a lot of attention on toxic agents which are produce during cooking process^5^. Method of cooking is a considerable source of toxicants that may have adverse health effects^6^. Type of dietary pattern, frequency of consumption and the kind of cooking method are most important determinants which appoint the level of risk of exposure to these agents. Investigators have shown that barbecued and charcoal grilled foods have high concentration of toxicants mostly because of fat and protein contents of the mentioned foods^5^. Grilled foods are becoming more and more popular (usually due to being palatable and ease of cooking) both at home and in restaurants, so as mentioned above, especial attention must be given to foods which are produced by these methods because they have more carcinogenic compounds in comparison with the other methods^7^^,^^8^.

Regarding the fuel type, it should be noted that several heating sources are used for cooking worldwide, including wood or charcoal, coal, kerosene, gas, electrical energy, etc.^6^. Cooking of foods, especially kebab, by charcoal can be dangerous due to the formation of carcinogens^7^.

Human beings eat foods (a combination of different nutrients), and these nutrients definitely have synergetic effects on health parameters. Dietary pattern is a more extended expression which includes all different types of foods and nutrients, the way of their consumption, etc., and it is also a more powerful predictor of individual health and disease related to foods^9^^,^^10^. 

Kebab is one of the most popular foods in the Middle East, particularly in Iran. Red meat, especially lamb or veal, is the most common meat used in preparation of kebab. In Iran, there are about seven types of kebab, including Kubide, Juje, Bakhtiari, Soltani, Dande, Fish and Barg. They are different in preparation methods, additives and may be in the kind of meat consumed (red meat, fish, fowl, etc). Kebab may be served with rice (chelow kebab) or special kinds of breads such as Sangak or Lavash. Kermanshah is a province located in the west of Iran, and it seems likely that people living there are interest in the consumption of meat, especially red meat, in the form of kebab as described previously. To date, no study has conducted to assess this dietary pattern in Iran, especially in this province, and it is a unique presentation in this field. Since kebab is considered as a food containing carcinogenic compounds, so the purpose of this study was to assess the consumption pattern of kebab in a sample of Iranian adults and its relationship with demographic characteristics.

## MATERIALS AND METHODS

 This cross-sectional study was conducted between March and April 2015 on 705 Iranian adults who were living in Kermanshah, a province in west of Iran. Subjects were selected randomly from different districts of Kermanshah. The data were collected using a questionnaire which had been designed by academic members of Department of Nutrition at Kermanshah University of Medical Sciences. Since kebab is among the most popular Iranian foods and has a particular recipe, so there is not any international or standard questionnaire to measure the pattern of this food consumption. In this study, researchers prepared a questionnaire in which common patterns and pathophysiologic mechanisms of this pattern were considered. Validity and reliability of questionnaire were confirmed under supervision of several local nutritionists who were familiar with the dietary pattern of people in Kermanshah. We also conducted a pilot study to address potential problems of questionnaire (results of the pilot study have not been mentioned). This questionnaire had two components: the first part was about demographic characteristics of subjects and the second one consisted of questions about the pattern of kebab consumption. The questionnaire contained 21 questions, 9 of which were about demographic characteristics (age, job, level of education, address, etc.), and the rest was about kebab consumption patterns (frequency of intake, interest in the use of kebab, types of kebab, methods of cooking, place of consumption, amount of salt intake, etc.) . In the questionnaire, we asked participants “How much are you willing to eat kebab?”. To answer to the question, four options were considered: low, medium, high and very high. We also asked participants two questions regarding their desire to consume salt with kebab. If the answer was positive, then they were asked about the amount of intake salt (low, medium, high or very high). Study participants, in terms of levels of education, were categorized as follows: illiterate, elementary, cycle, diploma, bachelor or above. In terms of jobs, they were classified to the unemployed, free and governmental. Body mass index (BMI) was calculated as weight (in kg) divided by height (in m^2^). Data collection was conducted in the way of face to face interview by three trained dietitians. For statistical analysis, we assessed normal distribution of data by the Kolmogorov-Smirnov test. Data analysis was performed using SPSS Version 20. The results were expressed as mean ± SD. Student’s t-test, ANOVA and chi-square tests were performed to compare the study groups. A P-value less than 0.05 was considered significant.

## Results

 This study included 705 participants (51.3% were male and 48.7% were female) with a mean age of 35 ± 10 years. In the present study, there was no significant difference between the mean age of men and women (Characteristics of subjects are shown in Table 1). 

**Table 1:** Characteristics of the adults

Characteristics	Groups	N (%)
**Gender**	Female	343(48.7%)
	Male	362(51.3%)
**Education levels**	Low	139(19.7%)
	High	566(80.3%)
**Job**	Unemployed	178(25.2%)
	Self-employed	183(26%)
	Employee	344(48.8%)
**Marital status**	Single	176(25.1%)
	Married	521(73.9%)
	Others	8(1%)

**Figure 1:**
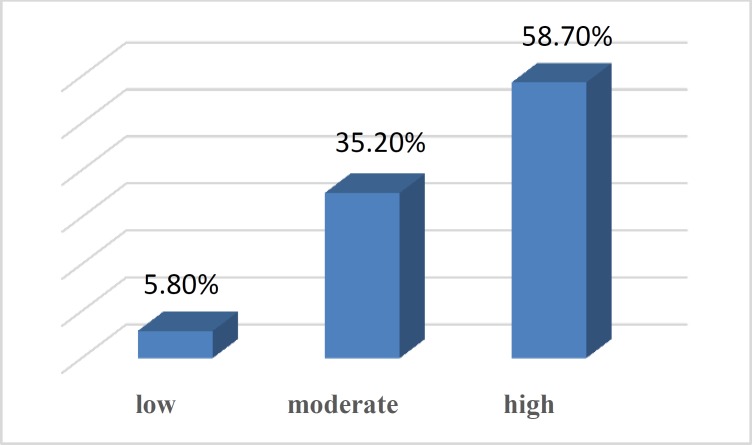
The rate of tendency to consume kebab

The results of statistical analysis showed that near to 60% of subjects had high tendency to consume kebab (Figure 1). 

The average of kebab consumption among the participants in this study was 4 times per a month, and this average in men was significantly more than in women (4.2±2.7 vs 3.8±2.6; p<0.03). Mean of BMI was more in men who had more tendency to consume kebab than those who had low tendency (25.3±3.7 vs 24.5±2.9; p<0.01).The relationship between age of subjects and their desire to consume kebab was not significant, but there was a statistically significant difference between gender of subjects and their desire to consume kebab (p<0.02). Men were more likely than women to consume kebab. More than 85% of participants in this study were interested in eating kebab with salt. We also found out a significant relationship between level of education and the tendency to consume kebab; participants with high level of education had more tendency to consume kebab than those having low level of education (p=0.02). The results obtained regarding the consumption of salt with kebab were different; the consumption of salt was higher among participants with low level of education in comparison to their more educated counterparts (p<0.033). Among the different kinds of kebabs, 39% and 37.4% of participants tended to consume chicken and kubideh kebab**,** respectively. Kubideh, as described previously, is a fatty meat mixed with onion and specific spices. These ingredients are mixed together, and then pressed around a skewer. 

 In this study, 93.9% of people used charcoal as a cooking fuel to prepare kebab. The results showed that 64.4% of subjects preferred to prepare and consume kebab at home. One of the questions asked from the participants was “what would be the food of your choice in the offered menu when you go to the restaurant for a meal?” and more than 60% selected kebab, 37.7% kubideh kebab with rice and 25.9% Jujeh kebab. In order to make Jujeh kebab, the chicken has to be marinated in onion, lemon juice, saffron , and salt before it goes to the grill. It is sometimes served with grilled tomato and pepper. 

## Discussion

 65.5% of main causes of death from non-communicable diseases are because of chronic disease such as diabetes, cancer, etc. Dietary pattern should receive more attention for being one of the most important risk factors for the above-mentioned diseases ^11^.

People living all over the world and also in different latitudes a country based on weather status, available food resources, various kinds of ethnic groups, etc. follow different dietary patterns, and this different dietary pattern have different effects on diseases. In Iran, kebab is served in all restaurants across the country.

Some types of carcinogenic compounds, including heterocyclic amines (HA_s_) and polycyclic aromatic hydrocarbons (PAH_s_) are produced during such cooking methods which occur at high temperature (barbecue, grilling)^12^. Professor Sugimura discovered these compounds about 30 years ago^13^. To date, about 100 kinds of PAH_s_ have been recognized, and some of them are confirmed to be carcinogenic. As mentioned above, these compounds are produced during pyrolysis process of components like fats and direct heat. So cooking method and fat content of meats are two major determinants in a carcinogenic food ^14^.

HA_s_ are categorized into two main groups: ‘‘thermic HA_s_’’ and “pyrolytic HA_s_’’. Thermic HA_s_ are formed complex pathway named Maillard reaction at temperatures between 150 and 250 degree Celsius (°C) and pyrolytic HA_s_ are produced at temperatures above 250(°C)^5^^,^^15^. It has been demonstrated that smoke of barbecued foods can be harmful because inhalation and also dermal contact to PAH_s_ can expose human to these compounds^7^. 

Different cultures have different dietary patterns and cooking methods, so due to these differences, individuals may be exposed to different types of pathogenic compounds, including carcinogenic compounds5. For instance, reducing heating time can reduce Benzo[a]pyrene (BaP) content of foods^14^. Studies also demonstrated that cooked meats which contain high levels of heterocyclic amines can lead to an increased risk of colorectal^16^, pancreas ^17^^-^^18^ and prostate^19^^-^^20^ cancers. One study has shown that eating each 10 grams of well-down red meat is correlated with increasing risk of colorectal adenoma by 29% ^13^. In another study in Argentina, consumption of all kinds of grilled meats had a significant positive relationship with colorectal cancer ^21^. 

According to the results of this study, more than half of those surveyed had a high tendency to consume kebab and remarkable percentage of whom were using charcoal as a cooking fuel to prepare barbecue. The noteworthy point is that the tendency and kebab consumption patterns were irrelevant to most of socio-economic factors (gender, Job…). In this study, individuals with high level of education were more likely to use barbecue than those who had low level of education and they followed an unhealthy pattern (Use larger amount of salt, etc) to consume kebab. This consumption pattern can be related to being pleasant and dainty of barbecue, easy access and ease of cooking. As noted above, most of the participants preferred to consume kebab at home and, in restaurants, they preferred to order kebab . 

## CONCLUSION

 Based on the findings of this study, it can be said that cooking barbecue in Kermanshah doesn’t follow the healthy pattern, and according to the mentioned mechanisms it can be considered as a powerful risk factor in development of cancer that needs to be modified. 

However, to derive an absolute conclusion about the association between cancer and the use of barbecues such as kebab we need to do more researches. Most scientists and nutritionists in industrialized countries have recommended decreasing consumption of barbecue^14^.

To minimize these compounds in diet, it is recommended to avoid direct exposure of meat to cooking surface (exactly similar to what happens in kebab preparation), and reduce cooking times to decrease HA_s_ and PAH_s_ contents. Cooking meat, especially by using a microwave, can reduce carcinogenic compounds. Moving and turning meat during cooking process can reduce the formation of PAH_s_ compared with methods in which meat is not stirred during cooking. When making barbecue, it should be noted that high temperatures will lead to the formation of carcinogenic compounds in barbecue^22^. We also recommend using large amounts of fruits and vegetables because they are rich in fiber, vitamins, minerals, antioxidants and photochemical, which help to protect the body against carcinogenic compounds.
